# *Troglostrongylus brevior*: a new parasite for Romania

**DOI:** 10.1186/s13071-017-2551-4

**Published:** 2017-12-06

**Authors:** Georgiana Deak, Angela Monica Ionică, Andrei Daniel Mihalca, Călin Mircea Gherman

**Affiliations:** Department of Parasitology and Parasitic Diseases, University of Agricultural Sciences and Veterinary Medicine, Calea Mănăștur 3–5, 400372 Cluj-Napoca, Romania

**Keywords:** *Troglostrongylus brevior*, *Felis silvestris*, Romania

## Abstract

**Background:**

The genus *Troglostrongylus* includes nematodes infecting domestic and wild felids. *Troglostrongylus brevior* was described six decades ago in Palestine and subsequently reported in some European countries (Italy, Spain, Greece, Bulgaria, and Bosnia and Herzegovina). As the diagnosis by the first-stage larvae (L1) may be challenging, there is a possibility of confusion with *Aelurostrongylus abstrusus*. Hence, the knowledge on the distribution of this neglected feline parasite is still scarce. The present paper reports the first case of *T. brevior* infection in Romania. In July 2017, a road-killed juvenile male *Felis silvestris*, was found in in Covasna County, Romania. A full necropsy was performed and the nematodes were collected from the trachea and bronchioles. Parasites were sexed and identified to species level, based on morphometrical features. A classical Baermann method was performed on the lungs and the faeces to collect the metastrongyloid larvae. Genomic DNA was extracted from an adult female nematode. Molecular identification was accomplished with a PCR assay targeting the ITS2 of the rRNA gene.

**Results:**

Two males and one female nematodes were found in the trachea and bronchioles. They were morphologically and molecularly identified as *T. brevior*. The first-stage larvae (L1) recovered from the lung tissue and faeces were morphologically consistent with those of *T. brevior*. No other pulmonary nematodes were identified and no gross pulmonary lesions were observed.

**Conclusions:**

This paper represents the first report of *Troglostrongylus brevior* infection in Romania, so far representing the second northernmost location for this genus in Europe. The diversity of species infecting wild and domestic felids and the differences regarding the clinical significance of these nematodes highlight the need for a more intense surveillance and proper diagnosis of feline lungworm infections, especially in countries where more species were demonstrated to be present. Furthermore, an increased awareness between clinicians is needed for a correct diagnostic approach to feline lungworm diagnosis.

## Background

A wide variety of nematode species belonging to the family Metastrongyloidea are known to infect domestic and wild felids. Among them, *Aelurostrongylus abstrusus* (Strongylida: Angiostrongylidae) has been considered for a long time to be the only metastrongyloid species parasitic in bronchi, bronchioles and alveoli of felids [1]. However, other species, such as *Troglostrongylus brevior*, *T. subcrenatus* (Strongylida: Crenosomatidae) and *Oslerus rostratus* (Strongylida: Filaroididae) have been reported more recently to cause respiratory diseases in cats [2–4]. The genus *Troglostrongylus* includes nematodes infecting domestic cats (*Felis silvestris catus*), wildcats (*Felis silvestris silvestris*, *Felis silvestris lybica*), leopards (*Panthera pardus*), tigers (*Panthera tigris*) and bobcats (*Lynx rufus*) [3]. *Troglostrongylus brevior* was described six decades ago in the bronchi of *F. s. lybica* and *F. chaus* in Palestine and reported more recently in domestic cats from Spain and Italy [3, 5, 6]. Since then, *T. brevior* has been found in recent years in domestic cats in Italy [7], Greece [8], Bulgaria [7], Spain [9], Cyprus [10], but also in *Lynx lynx* in Bosnia and Herzegovina [11]. The two species, *T. brevior* and *Ae. abstrusus* share a similar biology (indirect life-cycle, using intermediate and paratenic hosts); the diagnosis by the first-stage larvae (L1) may be challenging, which seems to have caused confusion between *T. brevior* and *Ae. abstrusus* for a long time [2, 12]. In contrast, adult nematodes inhabit specific respiratory tracts and they are clearly distinct morphologically. Adults of *Ae. abstrusus* are localized in the pulmonary tissue, forming sub-pleural nodules, while adults of *T. brevior* inhabit the trachea, bronchi and bronchioles [2, 3, 6]. *Troglostrongylus brevior* infection is more common in kittens and young cats, producing severe respiratory distress or being lethal, while reports of fatal *Ae. abstrusus* infection are unusual [2]. However, except a few large scales studies in domestic [7] and wildcats [13], the knowledge on the distribution of this neglected feline parasite is still scarce.

The present paper reports the first case of *T. brevior* infection in Romania, bringing proof for its broader geographic distribution in Europe.

## Methods

### Sample origin and lungworms collection

In July 2017, a road-killed juvenile male *Felis silvestris* in good body condition was collected near Crasna village in Covasna County (45.585185°N, 26.155503°E, 669 m altitude), Romania. The animal was identified based on morphological and morphometrical characteristics as either a wildcat or a hybrid [14] and the approximate age was established by teeth examination [15]. A full necropsy was performed and the trachea and the lungs were extracted and examined for the presence of parasites. The entire respiratory tract (trachea, bronchi and bronchioles) was longitudinally opened using scissors and observed under a stereomicroscope for the presence of parasites [6, 16]. Nematodes collected from the trachea and bronchioles were washed in saline solution and temporarily mounted on microscope slides, examined, measured and photographed, using an optical microscope (Olympus BX51; Soft Imaging solution GMBH LG20, Munster, Germany). Parasites were sexed and identified to species level, based on morphometrical features [5]. Small pieces from a female nematode were fixed and stored in 70% ethanol. A classical Baermann method [17] was performed on the lungs and the faeces to collect the metastrongyloid larvae (L1).

### Molecular analysis and species identification

Genomic DNA was extracted from an adult female nematode, using a commercial kit (Isolate II Genomic DNA Kit, Bioline, London, UK), according to the manufacturer’s instructions. Molecular identification was accomplished with a PCR assay, targeting the internal transcribed spacer 2 (ITS2, ~500 bp) of the rRNA gene, using primers and protocols available in the literature [18, 19]. Amplicons were purified using silica membrane spin columns (QIAquick PCR Purification Kit, Qiagen, Hilden, Germany) and then sequenced by Macrogen Europe (Amsterdam, Netherlands). The sequence was compared to those available in the GenBank by Basic Local Alignment Tool (BLAST) analysis. Species identification (adults and larvae) was based upon morphological characteristics, correlated with molecular analysis [1, 5, 6, 20].

## Results

### Morphology and morphometry of *Troglostrongylus brevior*

**Fig. 1:**
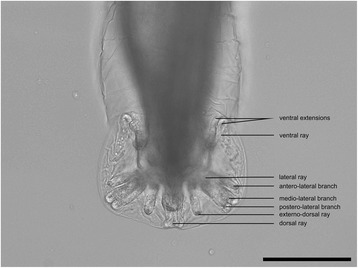
Characteristic copulatory bursa in male *Troglostrongylus brevior*. *Scale-bar*: 100 μm

Nematodes were found in the trachea and bronchioles (two males and one female) and were morphologically identified as *T. brevior*. The males were 7.73 mm and 7.76 mm long, with 0.29 mm and 0.31 mm maximum width, respectively, presenting a folded cuticle. The length of oesophagus varied between 0.27 mm and 0.30 mm, and the excretory pore was located at 107.45**–**113.13 μm from the cephalic end. The posterior end of males showed a well-developed copulatory bursa (Fig. 1), without a clear delineation of the median and lateral lobes and several distinct rays: dorsal, externo-dorsal, lateral and ventral. The dorsal ray was elongated and showed four apical papillae. The externo-dorsal ray was shorter than the dorsal ray, well-separated by dorsal and lateral rays and had a single extension. The lateral ray was divided into three branches: antero-lateral, medio-lateral and postero-lateral; the second and third were partially joined from the basis along half of their length. The ventral ray was medium sized compared to the other rays and distally split into two small extensions. The spicules were equally calibrated, measuring 0.64 mm, and 0.66 mm, respectively. Due to partial destruction during the removal from the lung tissue, the female nematode could not be measured.

The first-stage larvae (L1) recovered from the lung tissue and faeces were morphologically consistent with those of *T. brevior* (average length of 0.34 mm, average width of 0.02 mm) with a sub-terminal oral opening and a pointed tail with a pronounced dorsal spine and a less deep ventral incision.

No other pulmonary nematodes or intestinal parasites were found and no gross pulmonary lesions were observed.

### Molecular analysis

The BLAST analysis of the ITS2 sequence (accession number MF997544) revealed a 100% nucleotide similarity to *Troglostrongylus brevior* from Sardinia (KF241978.1).

## Discussion

Wildcats are considered to be an important reservoir and also the natural hosts for *Troglostrongylus* spp. [16]. *Troglostrongylus brevior* was originally described from wild felids in Palestine [5] and since then, the nematode has been found predominantly in countries where population of wildcats are present [7, 8, 11]. *Troglostrongylus* infections in domestic cats were reported for the first time in Europe, after 60 years from the first description in wildcats [3, 6, 9]. This may be due to the misdiagnosed cases of *Troglostrongylus* in domestic cats, as the first-stage larvae morphologically resemble those of *Ae. abstrusus* [6] and most of the diagnoses are based on coproscopy [7, 21–24]. However, *T. brevior* might be emerging in domestic cats, as a result of an increased interaction between wild and domestic carnivores, due to urbanization by land-clearing that forces wildlife to move into new areas, such as suburbs. The few cases of troglostrongylosis in domestic cats are characterized by severe and fatal clinical outcome. Clinical signs reported in young domestic cats include general respiratory signs, coughing, tachypnea, dyspnoea, polypnoea and depression, while in adult animals the infection is subclinical [2].

In the cat from the present study no gross lesions were noted, possibly highlighting the co-evolution of this parasite-host system. However, the absence of the pathological changes may be related also to the small number of adult parasites identified. In domestic cats, *T. brevior* produces lung oedema, haemorrhages, emphysema and catarrhal exudate in the airways [6, 25, 26]. *Troglostrongylus brevior*, *Ae. abstrusus* and *Angiostrongylus chabaudi* share a similar biology and all are using molluscs as intermediate hosts in their life-cycle [1, 5, 27], sometimes even occupying the same ecological niche and evolving as mixed infections in the same host [28]. *Troglostrongylus brevior* was found in association with *Ae. abstrusus*, rather than in single infections [9, 20, 29, 30] or also in association with *An. chabaudi*, a feline vascular parasite [28]. In the present study the infection was monospecific, as neither *Ae. abstrusus* nor *An. chabaudi* were present. In the last 10 years, carnivore lungworm infections were reported as spreading into new areas and in some countries the infection became emerging [31–38]. However, this might be also a result of more intense surveillance rather than a true emergence.

The diversity of species infecting wild and domestic felids and the differences regarding the clinical significance of these nematodes highlight the need for a more intense surveillance and proper diagnosis of feline lungworm infections, especially in countries where more species were demonstrated to be present [8]. Furthermore, an increased awareness between clinicians is needed for a correct diagnostic approach to feline lungworm diagnosis.

## Conclusions

This paper represents the first report of *Troglostrongylus brevior* infection in Romania, so far representing the second northernmost location for this genus in Europe.

## References

[1] Anderson RC (2000). The superfamily Metastrongyloidea. Nematode parasites of vertebrates. Their development and transmission.

[2] Traversa D, Di Cesare A (2013). Feline lungworms: what a dilemma. Trends Parasitol.

[3] Brianti E, Giannetto S, Dantas-Torres F, Otranto D (2014). Lungworms of the genus *Troglostrongylus* (Strongylida: Crenosomatidae): neglected parasites for domestic cats. Vet Parasitol.

[4] Brianti E, Gaglio G, Napoli E, Falsone L, Giannelli A, Annoscia G (2014). Feline lungworm *Oslerus rostratus* (Strongylida: Filaridae) in Italy: first case report and histopathological findings. Parasitol Res.

[5] Gerichter CB (1949). Studies on the nematodes parasitic in the lungs of Felidae in Palestine. Parasitology.

[6] Brianti E, Gaglio G, Giannetto S, Annoscia G, Latrofa MS, Dantas-Torres F (2012). *Troglostrongylus brevior* and *Troglostrongylus subcrenatus* (Strongylida: Crenosomatidae) as agents of broncho-pulmonary infestation in domestic cats. Parasit Vectors.

[7] Giannelli A, Capelli G, Hinney B, Joachim A, Losson B, Kirkova Z (2017). Epidemiology, diagnosis and treatment of lungworm and gastrointestinal parasitic infections in cats: an European perspective. Int J Parasitol.

[8] Diakou A, Di Cesare A, Barros LA, Morelli S, Halos L, Beugnet F, Traversa D (2015). Occurrence of *Aelurostrongylus abstrusus* and *Troglostrongylus brevior* in domestic cats in Greece. Parasit Vectors.

[9] Jefferies R, Vrhovec MG, Wallner N, Catalan DR (2010). *Aelurostrongylus abstrusus* and *Troglostrongylus* sp. (Nematoda: Metastrongyloidea) infections in cats inhabiting Ibiza, Spain. Vet Parasitol.

[10] Diakou A, Sofroniou D, Di Cesare A, Kokkinos P, Traversa D. Occurrence and zoonotic potential of endoparasites in cats of Cyprus and a new distribution area for *Troglostrongylus brevior*. Parasitol Res. 2017. doi:10.1007/s00436-017-5651-3. 10.1007/s00436-017-5651-3 29034414

[11] Alić A, Traversa D, Duscher GG, Kadrić M, Di Cesare A, Hodžić A (2015). *Troglostrongylus brevior* in an Eurasian lynx (*Lynx lynx*) from Bosnia and Herzegovina. Parasit Vectors.

[12] Otranto D, Brianti E, Dantas-Torres F (2013). *Troglostrongylus brevior* and a nonexistent ‘dilemma’. Trends Parasitol.

[13] Napoli E, Anile S, Arrabito C, Scornavacca D, Mazzamuto MV, Gaglio G (2016). Survey on parasitic infections in wildcat (*Felis silvestris silvestris* Schreber, 1777) by scat collection. Parasitol Res.

[14] Krüger M, Hertwig ST, Jetschke G, Fischer MS (2009). Evaluation of anatomical characters and the question of hybridization with domestic cats in the wildcat population of Thuringia, Germany. J Zool Syst Evol Res.

[15] Condé B, Schauenberg P (1978). Replacement des canines chez le chat forestier *Felis silvestris* Schreb. Rev Suisse Zool.

[16] Falsone L, Brianti E, Gaglio G, Napoli E, Anile S, Mallia E (2014). The European wildcats (*Felis silvestris silvestris*) as spreaders of *Troglostrongylus brevior* (Strongylida: Crenosomatidae) lungworms. Vet Parasitol.

[17] Willcox HP, Coura JR. A new design of the Baermann, Moraes, Coutinho’s technique for the isolation of nematode larva. Mem Inst Oswaldo Cruz. 1989;84:563–5. (In Portuguese). 10.1590/s0074-02761989000400015 2487451

[18] Gasser RB, Chilton NB, Hoste H, Beveridge I (1993). Rapid sequencing of rDNA from single worms and eggs of parasitic helminths. Nucleic Acids Res.

[19] Caldeira RL, Carvalho OS, Mendonça CL, Graeff-Teixeira C, Silva MC, Ben R (2003). Molecular differentiation of *Angiostrongylus costaricensis*, *A. cantonensis*, and *A. vasorum* by polymerase chain reaction-restriction fragment length polymorphism. Mem Inst Oswaldo Cruz.

[20] Tamponi C, Varcasia A, Brianti E, Pipia AP, Frau V, Pinna Parpaglia ML (2014). New insights on metastrongyloid lungworms infecting cats of Sardinia. Italy. Vet Parasitol.

[21] Payo-Puente P, Botelho-Dinis M, Carvaja Urueña AM, Payo-Puente M, Gonzalo-Orden JM, Rojo-Vazquez FA (2008). Prevalence study of the lungworm *Aelurostrongylus abstrusus* in stray cats of Portugal. J Feline Med Surg.

[22] Traversa D, Lia RP, Iorio R, Boari A, Paradies P, Capelli G (2008). Diagnosis and risk factors of *Aelurostrongylus abstrusus* (Nematoda, Strongylida) infection in cats from Italy. Vet Parasitol.

[23] Mircean V, Titilincu A, Vasile C (2010). Prevalence of endoparasites in household cat (*Felis catus*) populations from Transylvania (Romania) and association with risk factors. Vet Parasitol.

[24] Knaus M, Kusi I, Rapti D, Xhaxhiu D, Winter R, Visser M, Rehbein S (2011). Endoparasites of cats from the Tirana area and the first report on *Aelurostrongylus abstrusus* (Railliet, 1898) in Albania. Wien Klin Wochenschr.

[25] Giannelli A, Passantino G, Ramos RA, LoPresti G, Lia RP, Brianti E (2014). Pathological and histological findings associated with the feline lungworm *Troglostrongylus brevior*. Vet Parasitol.

[26] Traversa D, Romanucci M, Di Cesare A, Malatesta D, Cassini R, Iorio R (2014). Gross and histopathological changes associated with *Aelurostrongylus abstrusus* and *Troglostrongylus brevior* in a kitten. Vet Parasitol.

[27] Colella V, Cavalera MA, Deak G, Tarallo VD, Gherman CM, Mihalca AD, Otranto D. Larval development of *Angiostrongylus chabaudi*, the causative agent of feline angiostrongylosis, in the snail *Cornu aspersum*. Parasitology. 2017;144:1922–30. 10.1017/S0031182017001433 28805181

[28] Traversa D, Lepri E, Veronesi F, Paoletti B, Simonato G, Diaferia M, Di Cesare A (2015). Metastrongyloid infection by *Aelurostrongylus abstrusus*, *Troglostrongylus brevior* and *Angiostrongylus chabaudi* in a domestic cat. Int J Parasitol.

[29] Di Cesare A, di Regalbono FA, Tessarin C, Seghetti M, Iorio R, Simonato G, Traversa D (2014). Mixed infection by *Aelurostrongylus abstrusus* and *Troglostrongylus brevior* in kittens from the same litter in Italy. Parasitol Res.

[30] Di Cesare A, Di Francesco G, di Regalbono FA, Eleni C, de Liberato C, Marruchella G (2015). Retrospective study on the occurrence of the feline lungworms *Aelurostrongylus abstrusus* and *Troglostrongylus* spp. in endemic areas of Italy. Vet J.

[31] Helm JR, Morgan ER, Jackson MW, Wotton P, Bell R (2010). Canine angiostrongylosis: an emerging disease in Europe. J Vet Emerg Crit Care (San Antonio).

[32] Helm J, Roberts L, Jefferies R, Shaw SE, Morgan ER (2015). Epidemiological survey of *Angiostrongylus vasorum* in dogs and slugs around a new endemic focus in Scotland. Vet Rec.

[33] Taylor CS, Garcia Gato R, Learmount J, Aziz NA, Montgomery C, Rose H (2015). Increased prevalence and geographic spread of the cardiopulmonary nematode *Angiostrongylus vasorum* in fox populations in great Britain. Parasitology.

[34] Diakou A, Psalla D, Migli D, Di Cesare A, Youlatos D, Marcer F, Traversa D (2016). First evidence of the European wildcat (*Felis silvestris silvestris*) as definitive host of *Angiostrongylus chabaudi*. Parasitol Res.

[35] Gherman CM, Ionică AM, D’Amico G, Otranto D, Mihalca AD. *Angiostrongylus chabaudi* (Biocca, 1957) in wildcat (*Felis silvestris silvestris, S*) from Romania. Parasitol Res. 2016;115:2511–7. 10.1007/s00436-016-5032-3 27106235

[36] Gherman CM, Deak G, Matei IA, Ionică AM, D’Amico G, Taulescu M, et al. A rare cardiopulmonary parasite of the European badger, *Meles meles*: first description of the larvae, ultrastructure, pathological changes and molecular identification of *Angiostrongylus daskalovi* Janchev & Genov, 1988. Parasit Vectors. 2016;9:423. 10.1186/s13071-016-1718-8 PMC4969667 27485118

[37] Giannelli A, Kirkova Z, Abramo F, Latrofa MS, Campbell B, Zizzo N (2016). *Angiostrongylus chabaudi* in felids: new findings and a review of the literature. Vet Paras.

[38] Deak G, Gherman CM, Ionică AM, Vezendan AD, D’Amico G, Matei IA (2017). *Angiostrongylus vasorum* in Romania: an extensive survey in red foxes, *Vulpes vulpes*. Parasit Vectors.

